# Comprehensive Survival Analysis of Alveolar Echinococcosis Patients, University Hospital Zurich, Zurich, Switzerland, 1973–2022

**DOI:** 10.3201/eid3105.241608

**Published:** 2025-05

**Authors:** Ansgar Deibel, Yanick Kindler, Rubens Mita, Soleen Ghafoor, Cordula Meyer zu Schwabedissen, Barbara Brunner-Geissmann, Alexander Schweiger, Felix Grimm, Michael Reinehr, Achim Weber, Cäcilia S. Reiner, Andreas E. Kremer, Henrik Petrowsky, Pierre-Alain Clavien, Peter Deplazes, Stefanie von Felten, Beat Müllhaupt

**Affiliations:** University Hospital Zurich, Zurich, Switzerland (A. Deibel, Y. Kindler, S. Ghafoor, C. Meyer zu Schwabedissen, B. Brunner-Geissmann, M. Reinehr, A. Weber, C.S. Reiner, A.E. Kremer, H. Petrowsky, P.-A. Clavien, B. Müllhaupt); University of Zurich, Zurich (R. Mita, F. Grimm, P. Deplazes, S. von Felten); Cantonal Hospital Zug, Zug, Switzerland (A. Schweiger)

**Keywords:** Alveolar echinococcosis, *Echinococcus multilocularis*, survival, relative survival, mortality, surgery, resection, albendazole, mebendazole, benzimidazole, parasites, Zurich, Switzerland,

## Abstract

Alveolar echinococcosis (AE) is a zoonotic disease of increasing concern worldwide. Before benzimidazole drug therapy, 10-year death rates were 90% without surgical resection. In unresectable patients, long-term benzimidazole therapy is highly effective in stabilizing the disease course. We performed a retrospective study of 334 AE patients treated at the University Hospital Zurich, Zurich, Switzerland, during 1973–2022. Annual diagnoses increased over time, and more cases were detected by chance at earlier stages. Ninety patients died, mostly from causes unrelated to AE. Relative survival of AE patients compared with the population of Switzerland demonstrated a steady decrease 5 years after diagnosis. Patient age at diagnosis was the primary variable associated with overall survival. In a propensity-score matched survival analysis, early curative surgery was associated with overall improvement but not AE-specific survival. We conclude that survival of patients with AE is limited by non-AE causes and that early curative surgery does not improve AE-specific survival.

Alveolar echinococcosis (AE) is an orphan zoonosis caused by the metacestode stage of the fox tapeworm, *Echinococcus multilocularis*. This parasite is endemic across large parts of the Northern Hemisphere, including Switzerland, Germany, and France (*1*). Although rare, AE is of increasing concern because of rising incidences (*2*–*6*). Previously nonendemic regions such as North America and eastern central Europe are reporting an increasing number of AE patients (*7*–*10*). Proposed explanations for this phenomenon include the habitat expansion of a growing fox population, an increased use of imaging in healthcare, and a more susceptible population (*4*,*5*,*11*).

AE is a silently progressing and infiltrative disease that primarily affects the liver and can become symptomatic through mass effect and occlusion of bile ducts or blood vessels (*12*). Various complications can occur, such as obstructive jaundice, cholangitis, portal vein occlusion or thrombosis, or secondary Budd Chiari syndrome with or without portal hypertension (*13*). On occasion, distant metastasis is observed (*12*). For staging of the disease the PNM classification (parasite location in the liver, neighboring organ involvement, metastasis) was proposed (*14*).

Without adequate treatment, 90% of AE patients died within 10 years of disease onset (*15*). Cure can only be achieved through complete resection and adjuvant benzimidazole drug recurrence prophylaxis (*16*). Curative resection is often not possible because of advanced disease (*16*). The use of palliative surgery was abandoned in the early 2000s because of a lack of survival benefit over benzimidazole drug therapy alone (*17*). Liver transplantation is associated with frequent disease recurrence and remains a rescue measure in select cases (*18*). In inoperable AE cases, long-term benzimidazole drug therapy is highly effective at stopping disease progression (*19*). Treating icteric patients because of biliary obstruction with benzimidazole drugs alone, rather than performing biliary tract intervention, might be as effective and safer (*20*). Today, selected inoperable patients can be considered for treatment discontinuation (*21*,*22*).

The life expectancy of AE patients has increased since benzimidazole drug therapy was introduced (*19*,*23*). Excess deaths caused by AE were reported to be highest in the first 2 years after diagnosis (*23*). The main death risk was attributed to hilar involvement of the AE and the age of patients when AE was diagnosed (*19*,*23*). Radical surgery and benzimidazole drug therapy have improved overall survival of AE patients (*19*,*23*).

The aim of this study was to assess changes in the clinical manifestation, treatment, and survival of AE patients treated at the University Hospital Zurich, Zurich, Switzerland, over a 50-year period. This study followed the Strengthening the Reporting of Observational Studies in Epidemiology statement checklist.

## Patients and Methods

### Patients

The Zurich Echinococcosis Cohort Study was launched in November 2020 after receiving ethical approval (Business Administration System for Ethics Committees approval no. 2020-00495). The study included all patients who underwent consultations for AE at Zurich University Hospital from 1973–2022, identified by the hospital’s electronic system and an AE cohort registry (*2*,*19*,*24*). We obtained informed consent during outpatient visits or by letter; for deceased patients, consent was waived by the ethics committee. We sourced clinical data from the old cohort registry and reviewed from both archived and electronic patient records.

### AE Diagnosis, Staging, Symptoms, and Complications

We classified AE diagnoses according to World Health Organization criteria as possible (imaging finding or positive serologic test), probable (imaging finding confirmed by 2 serologic tests), and definitive (confirmation through histopathologic test or PCR) (*14*). For staging AE, we applied the PNM classification through review of available imaging data at diagnosis (*14*). When no computed tomography or magnetic resonance imaging images were available for review, we cross-verified staging data entries from the cohort registry database with available imaging reports, which a radiologist with experience in AE imaging corrected in case of conflicting results (n = 10). We recorded the presence of any AE-associated symptoms, such as right upper quadrant pain, and the presence of any biliary, vascular, or infectious (nonbiliary) complications at the time of diagnosis. Biliary complications comprised biliary tract occlusion with jaundice or cholangitis. Vascular complications included portal vein, liver vein, or inferior vena cava occlusion or thrombosis with signs of portal-hypertension or inferior vena cava obstruction, which included the presence of ascites, esophageal varices, or lower leg edema. We defined infectious complications as AE-associated infections other than cholangitis, mainly cyst infections, empyema, or peritonitis.

### AE Treatment

We classified initial surgical resection of AE lesions by intent (curative or palliative) and by involvement of the liver (hepatic or nonhepatic). In case of curatively intended liver resection, we defined the resection margin as R0 or R1, depending on whether the AE lesion extended into the resection margin on histopathologic examination. We classified liver resection as mentioned in the case surgical report into segmentectomy, hemihepatectomy (segments I–IV or V–VIII), extended hemihepatectomy (segments I–VI or IV–VIII), or liver transplantation. We assessed the time from diagnosis to surgical intervention and classified into early (<12 months) and late (>12 months) resection. After curatively intended liver resection, we defined the detection of any new AE-typical lesions on repeat cross-sectional imaging as recurrence.

We recorded the initial benzimidazole drug therapy, the type (albendazole or mebendazole) of drug, and time from diagnosis to start of treatment. Curatively resected patients receive a postoperative recurrence prophylaxis with benzimidazole drugs for >2 years (*16*). In case of R1, palliative resection, or inoperable disease, benzimidazole drug therapy is continued indefinitely (*16*). In addition, if benzimidazole drug treatment was prematurely discontinued, we recorded the reason. We considered a structured treatment discontinuation in patients meeting the criteria of inactive disease, negative results on Em18/-EmII (*3*–*10*) serologic testing, and no metabolic activity of AE lesions on positron emission tomography–computed tomography (*22*). We considered any physician-initiated treatment discontinuation outside those criteria nonstructured. 

### Follow-Up and Survival Data

We recorded the date of last follow-up, follow-up duration, and clinical course of AE. Complete follow-up included patient history, imaging report (computed tomography or magnetic resonance imaging), serologic testing, and blood analysis until last contact or study closure date (by September 30, 2023). We recorded the occurrence of symptomatic events, including biliary complications (cholestasis or cholangitis) or vascular obstruction (ascites or variceal bleeding), cyst rupture, infection, or fistula formation. We considered patients cured if parasitic tissue was completely surgically removed and the disease did not reoccur during follow-up. In addition, we recorded date of death, obtained through patient charts or from the hospital administration that obtained the information through the national civil register, and cause of death. If the cause of death was not noted in the patient charts, we contacted the last treating physician or local hospital to provide that information. We grouped causes of death other than AE into 6 groups: malignant, cardiovascular, neurologic, hepatic (non-AE), infectious, and other diseases.

### Statistical Analysis

We conducted all analysis by using R (The R Project for Statistical Computing Team, https://www.r-project.org). We compared the survival of AE patients with the population of Switzerland by estimating relative survival curves and by using additive relative survival models as implemented in the R functions rs.surv and rsadd (with the method of expectation-maximization) from the relsurv package (*25*). We retrieved the life tables of the population of Switzerland from the Human Mortality Database (https://www.mortality.org).

To evaluate whether early curative surgery (within a year of diagnosis) improved overall and disease-specific survival compared with no, delayed, or palliative surgery, we used propensity score matching to balance baseline characteristics and applied Cox proportional hazards models on the matched set. We estimated the propensity score, the probability of receiving curative surgery within 1 year, by using logistic regression with patient age at AE diagnosis, year of diagnosis, PNM classification (MX was considered M0), incidental finding of AE, benzimidazole drug therapy within 1 year, and presence of AE complications at diagnosis as explanatory variables. We performed matching once by using 1:1 nearest neighbor matching on the propensity score without replacement and once by using 1:1 genetic matching, both targeting the average treatment effect on the treated, as implemented in the R package MatchIt (*26*).

## Results

### Patient Cohort

In total, we included 334 (93.8%) of 356 identified AE patients in the study (Table 1; Appendix Figure 1). Diagnosis was probable in 144 (43.1%) cases and definitive in 186 (55.7%) cases, according to World Health Organization criteria (*14*). Only in 4 patients (1.2%) was the diagnosis solely on the basis of typical imaging findings. The median patient age at diagnosis was 57.5 years of age, and there was a slight female predominance (57.5% female vs. 42.5% male). The liver was affected in most patients (97%, n = 331), whereas 96 (28.7%) patients demonstrated involvement of a neighboring organ, and 41 patients (12.3%) demonstrated distant metastasis (Appendix Table 1). AE manifested in a limited stage (I–II) in 135 (40.4%) patients and in an advanced stage (IIIa–IV) in 192 (57.6%) patients; in 7 patients (2.1%) AE could not be staged because of missing data. Most (60.2%, n = 201) patients had symptoms attributable to AE, whereas 127 (38.0%) patients had AE diagnosed incidentally. If complications were observed at diagnosis, biliary complications were reported most frequently (13.8%, n = 46), whereas vascular complications and nonbiliary infections occurred only in rare cases.

**Table 1:** Baseline characteristics of alveolar echinococcosis patients, University Hospital Zurich, Zurich, Switzerland, 1973–2022*

Baseline characteristics	Value
No. patients	334
Age at diagnosis, y, median (IQR)	57.5 (44.0–65.8)
Sex	
M	142 (42.5)
F	192 (57.5)
World Health Organization diagnosis criteria	
Possible	4 (1.2)
Probable	144 (43.1)
Definitive	186 (55.7)
Alveolar echinococcosis stage	
I	93 (27.8)
II	42 (12.6)
IIIa	50 (15.0)
IIIb	74 (22.2)
IV	68 (20.4)
Unclassified	7 (2.1)
Symptoms at diagnosis	
Yes	201 (60.2)
No	127 (38.0)
Missing	6 (1.8)
Complication at diagnosis	
Biliary	46 (13.8)
Vascular	8 (2.4)
Infectious	6 (1.8)
Missing	1 (0.3)

*Values are no. (%) except as indicated.

### Pursued Treatment and Observed Clinical Course

We observed different treatment strategies and clinical courses in our study (Table 2; Appendix Table 2). Surgical resection was performed in 151 (45.2%) patients after a median of 1 month. Only 10 patients had surgery >12 months after diagnosis. Twenty-five (16.6%) patients underwent an a priori palliative debulking resection. In 126 (83.4%) patients, a curative resection was intended, which was confirmed by histologic testing in 105 (83.3%) patients (R0 resection), whereas in 21 (16.7%) patients the resection margin was positive (R1 resection). Of the 126 patients who underwent curatively intended surgery, 13 suffered disease recurrence, most in cases of R1 resection (n = 8). All recurrences were in the liver, in 10 patients at the resection margin, whereas 3 patients showed new liver lesions. R1 resection resulted in a prolongation of benzimidazole drug therapy to a median of 79 months (median 26 months in R0 resected patients), and 10 patients were on drug therapy at last follow-up. Four patients with R0 resection received no recurrence prophylaxis because of suspected inactive disease. In all 4 patients, surgery was performed under the assumption of cancer metastasis to the liver, and AE infection was an incidental diagnosis. Serologic testing performed shortly after surgery was completely negative.

**Table 2:** Treatments used for alveolar echinococcosis patients, University Hospital Zurich, Zurich, Switzerland, 1973–2022*

Treatment	Value
No. patients	334
Surgical therapy	151 (45.2)
Time to surgery, mo, median (IQR)	1 (0–4)
Surgery aim	
Palliative	25 (7.5)
After >12 mo	5 (1.5)
Curative	126 (37.7)
After >12 mo	5 (1.5)
Resection margins	
R0	105 (31.4)
R1	21 (6.3)
Surgery type	
Segmentectomy	51 (15.3)
Hemihepatectomy	53 (15.9)
Extended hemihepatectomy	17 (5.1)
Liver transplantation	3 (0.9)
Nonhepatic surgery	2 (0.6)
Benzimidazole drug therapy	315 (94.3)
Time to benzimidazole start, mo, median (IQR)	1 (0–2)
>12 mo	17 (5.1)
Benzimidazole drug type	
Albendazole	241 (72.2)
Mebendazole	74 (22.2)
Clinical setting	
After curatively intended surgery	122 (36.5)
R0 margin	97 (29.0)
Duration, mo, median (IQR)	26 (23–31.5)
R1 margin	21 (6.3)
Duration, mo, median (IQR)	79 (44–152)
Palliative surgery and nonresected	212 (63.5)

*Values are no. (%) except as indicated.

Most commonly, patients underwent segmentectomy (n = 51) or hemihepatectomy (n = 53). Only 17 had an extended hemihepatectomy, and 3 patients underwent liver transplantation. Only 1 transplant was performed because of AE, whereas the other 2 had independent indications (hepatocellular and perihilar cholangiocellular carcinoma). Two patients underwent nonhepatic resection, 1 of an isolated cerebral lesion and 1 of an isolated lesion in the thoracic spine.

Benzimidazole drug therapy, mainly albendazole, was initiated in most patients (94.3%, n = 315) after a median of 1 month. In 212 (63.5%) patients who underwent palliative or no resection, albendazole was the mainstay of treatment. Only 15 nonresected patients did not receive benzimidazole drug therapy because of suspected inactive disease. Twenty (6.0%) patients who underwent palliative or no resection had a symptomatic progression event during follow-up. Twelve of those events were recurrent or new onset of cholestasis with or without cholangitis; 4 had portal-hypertensive complications including ascites or variceal bleeding, whereas another 5 had cyst rupture, infection, or fistula formation.

In 33 (9.9%) resected and nonresected patients, benzimidazole drug therapy was discontinued prematurely at various time points during follow-up, most commonly because of treatment-related adverse events (n = 10), followed by nonstructured discontinuations in R1 and palliative resected patients (n = 8), terminal illness other than AE (n = 7), patient choice (n = 4), intended pregnancy (n = 2), and other or unknown 2 (n = 2) (Appendix Table 2). Of all inoperable patients that discontinued benzimidazole drug therapy because of intolerance. only 1 patient died of AE, 95 months after treatment discontinuation (Table 3). Another 28 (8.5%) patients on long-term benzimidazole therapy underwent a structured treatment discontinuation after a median of 58.5 months.

**Table 3:** Characteristics of patients who died because of AE, University Hospital Zurich, Zurich, Switzerland, 1973–2022*

ID	Year diag	Age, y/sex	Stage	Symp	Comp	Surgery	Time to surgery, mo†	Aim	BMZ	Time to BMZ†	Survival time, mo†	Direct cause of death
1	1973	26/M	IV	Yes	None	Yes	37	Palliative, with biliodigestive anastomosis	Yes‡	94	384	Biliocutaneous fistula, recurrent biliary infections, secondary biliary cirrhosis
2	1975	57/M	IV	Yes	Vascular	Yes	0	Curative	Yes	80	238	Retroperitoneal recurrence, total IVC occlusion, GI hemorrhage
3	1975	54/F	IV	Yes	None	NA	NA	NA	Yes	77	104	Cerebral AE manifestation
4	1978	68/F	II	Yes	Biliary	Yes	0	Palliative, with biliodigestive anastomosis	Yes	0	35	Recurrent biliary infections, secondary biliary cirrhosis
5	1979	44/F	IIIa	Yes	Vascular	NA	NA	NA	Yes	0	281	ERCP for pancreatitis, decompensation, peritonitis
6	1980	61/F	IV	Yes	Biliary	NA	NA	NA	Yes	0	133	Secondary biliary cirrhosis
7	1982	67/M	IV	Yes	Vascular	NA	NA	NA	Yes	0	159	Variceal bleeding
8	1983	64/F	IIIb	Yes	Biliary	NA	NA	NA	Yes	0	53	Cholangitis with liver abscess
9	1983	47/F	II	Yes	None	Yes	0	Palliative	Yes	0	233	Progressive disease, secondary surgery, postoperative multiorgan failure
10	1988	67/M	IIIa	Yes	Biliary	Yes	0	Palliative	Yes	1	149	PTCD for cholangitis
11	2001	48/M	IV	Yes	None	Yes	0	Palliative, nonhepatic	Yes	0	193	Cerebral AE manifestation
12	2001	56/F	IIIb	Yes	Vascular	NA	NA	NA	Yes	0	187	Liver failure because of portal and liver vein thrombosis
13	2017	60/F	IV	Yes	Biliary	NA	NA	NA	Yes	0	70	PTCD for cyst infection with rupture and peritoneal dissemination

*AE, alveolar echinococcosis; BMZ, benzimidazole drug therapy; comp, complications at diagnosis; diag, diagnosed; ERCP, endoscopic retrograde cholangiopancreaticography; ID, identification number; IVC, inferior vena cava; PTCD, percutaneous transhepatic cholangiography and drainage; symp, symptoms at diagnosis; surg, surgery. †Time after diagnosis. ‡Discontinued after 195 months because of intolerance (95 months before death).

### Changes in Clinical Manifestation and Treatment over Time

**Figure 1:**
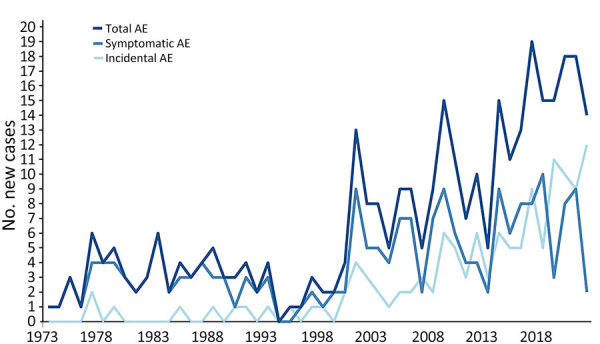
Total number of new, symptomatic, and incidental AE diagnosis by calendar year, University Hospital Zurich, Zurich, Switzerland, 1973–2022. AE, alveolar echinococcosis.

**Figure 2:**
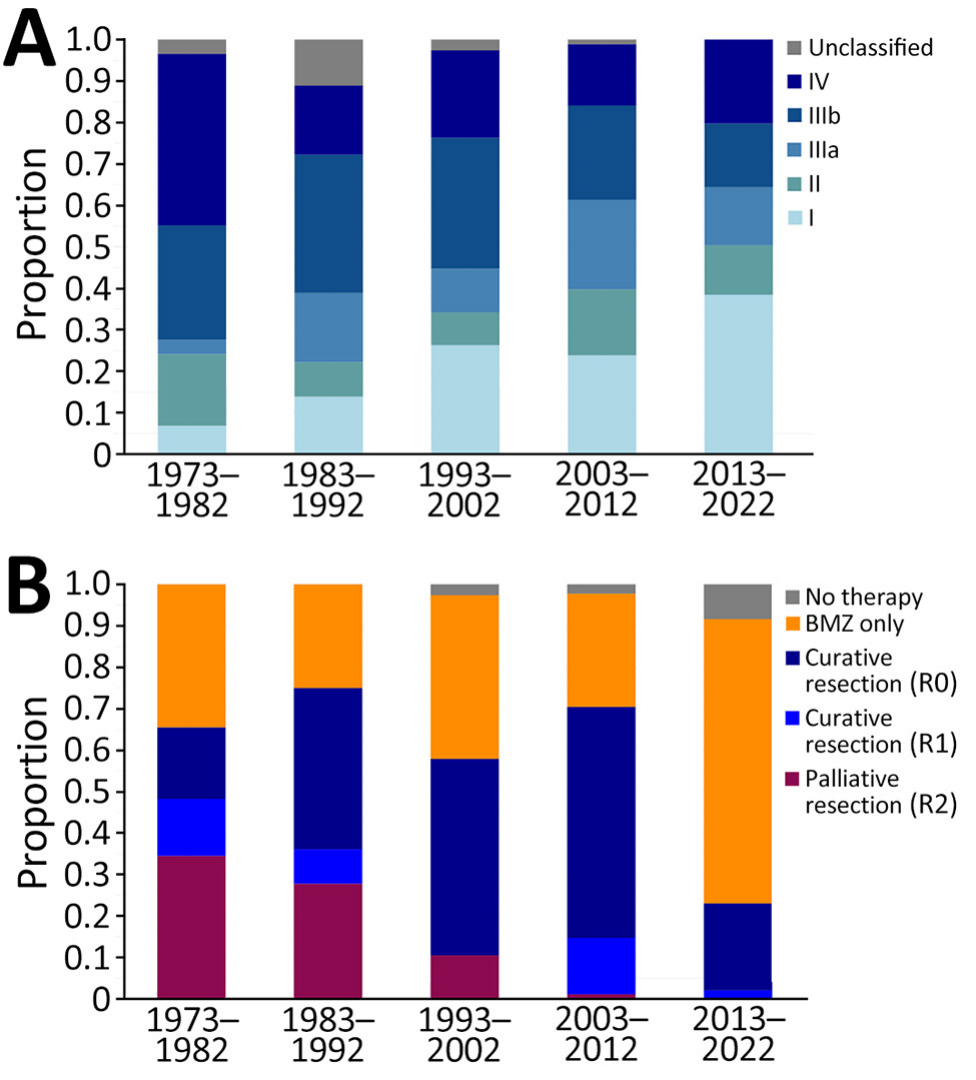
Stages and treatment of AE cases by decade, University Hospital Zurich, Zurich, Switzerland, 1973–2022. A) AE stages; B) treatment strategies. Although palliative surgery was discontinued in the early 2000s, curative surgery was less frequently pursued in the last decade of the study period. AE, alveolar echinococcosis; BMZ, benzimidazole drug therapy.

Over the decades of the study period, patient demographics remained similar (Appendix Table 4). Since 2000, a steady increase in new AE diagnosis per year was noted, with an increasing proportion attributable to incidental diagnosis (Figure 1). In fact, during the last 3 years of the study period, most of the patients with newly diagnosed AE had incidental diagnoses. Concordantly, a shift toward earlier AE stages was observed (Figure 2, panel A). With time, fewer patients underwent palliative resection; the last was performed in 2007 (Figure 2, panel B). Although the proportion of patients undergoing curatively intended resections rose at first, it decreased sharply in the last decade of the study period (Figure 2, panel B). Of interest, patients were also followed without treatment (Figure 2, panel B).

### Symptomatic versus Incidental Diagnosis

As expected, patients with incidental diagnoses showed more frequently a limited stage of the disease (Appendix Table 3). In contrast, those patients were slightly older at diagnosis than were patients with symptomatic disease (Appendix Table 3). Furthermore, patients with incidental diagnoses less often underwent surgery. The reported reason to forgo surgery was either disease inactivity, personal choice or presence of comorbidity, and, less frequently, disease extent. Benzimidazole drug treatment was also slightly less frequently initiated. Of interest, no AE related death was observed in the incidental group.

### Causes of Death

**Figure 3:**
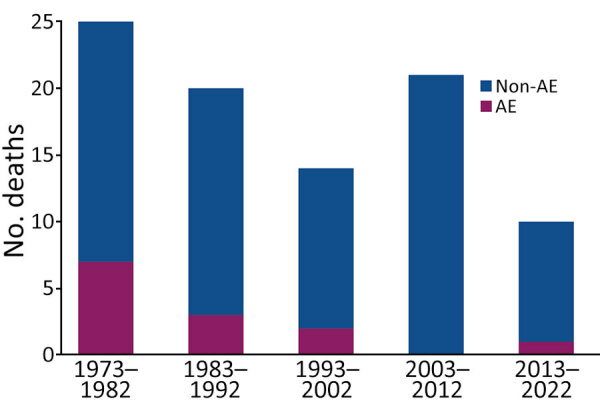
AE and non-AE associated causes of death for AE patients, by decade of AE diagnosis, University Hospital Zurich, Zurich, Switzerland, 1973–2022. AE associated death was observed more frequently in earlier decades of the study period. AE, alveolar echinococcosis.

**Figure 4:**
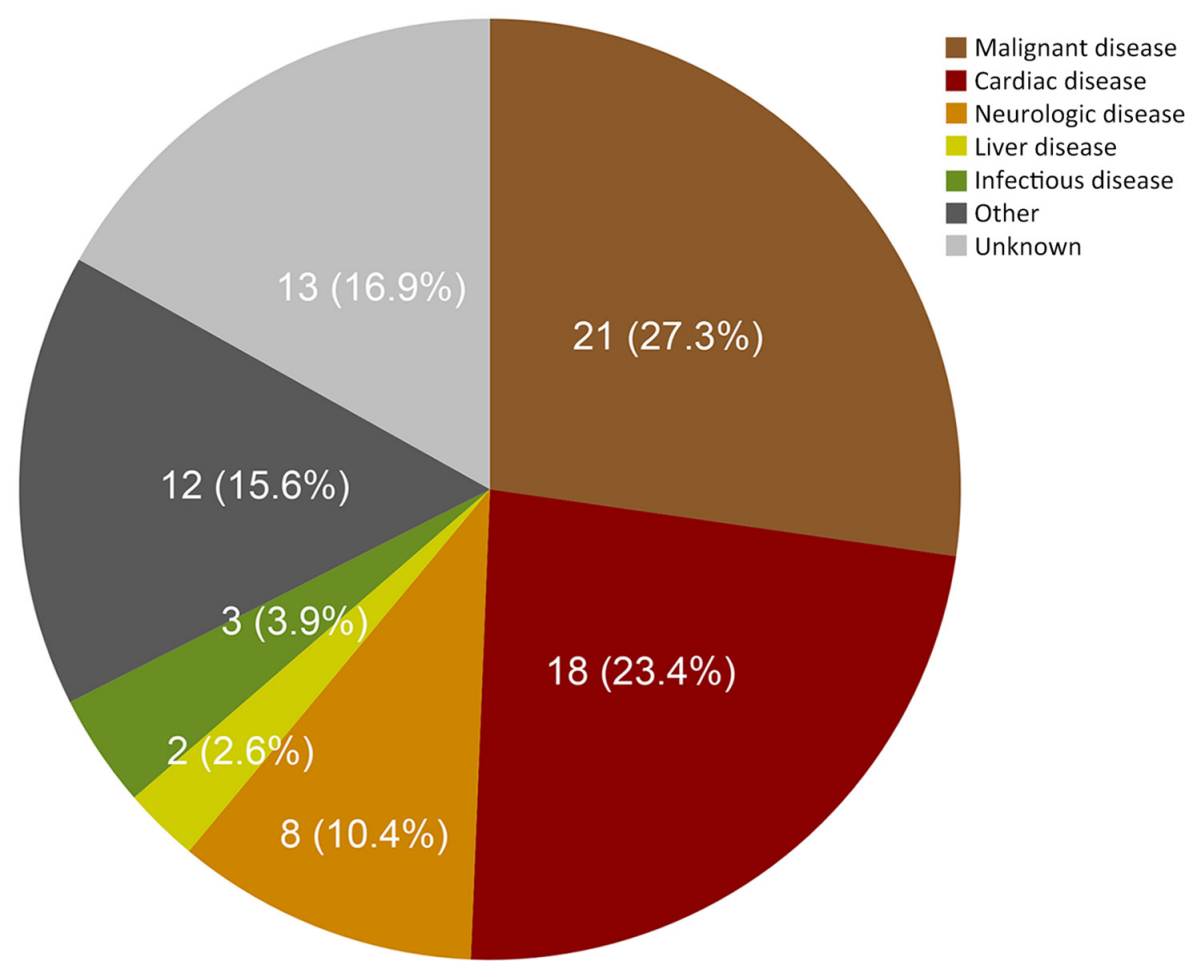
Causes of death other than alveolar echinococcosis in alveolar echinococcosis cases, University Hospital Zurich, Zurich, Switzerland, 1973–2022.

In total, 90 (26.9%) patients died after a median of 176 months, but causes of death were predominantly non-AE related (Figures 3, 4). AE-related death occurred more frequently in symptomatic patients whose AE was diagnosed in earlier decades (Figures 3, 4; Appendix Table 3). Most of those patients had biliary or vascular complications when AE was diagnosed (Table 3). Only 1 patient had undergone curatively intended resection, and 5 had palliative resections. The median time to AE-related death was 159 months.

The cause of death could not be determined in 13 patients. Their AE was diagnosed in an advanced stage but without any biliary, vascular, or infectious complication at diagnosis. The median age at death in this group was 89 years, and at last clinical visit, AE was considered as cured or stable, making AE unlikely as the cause of death (Appendix Table 5).

### Survival Analysis

**Figure 5:**
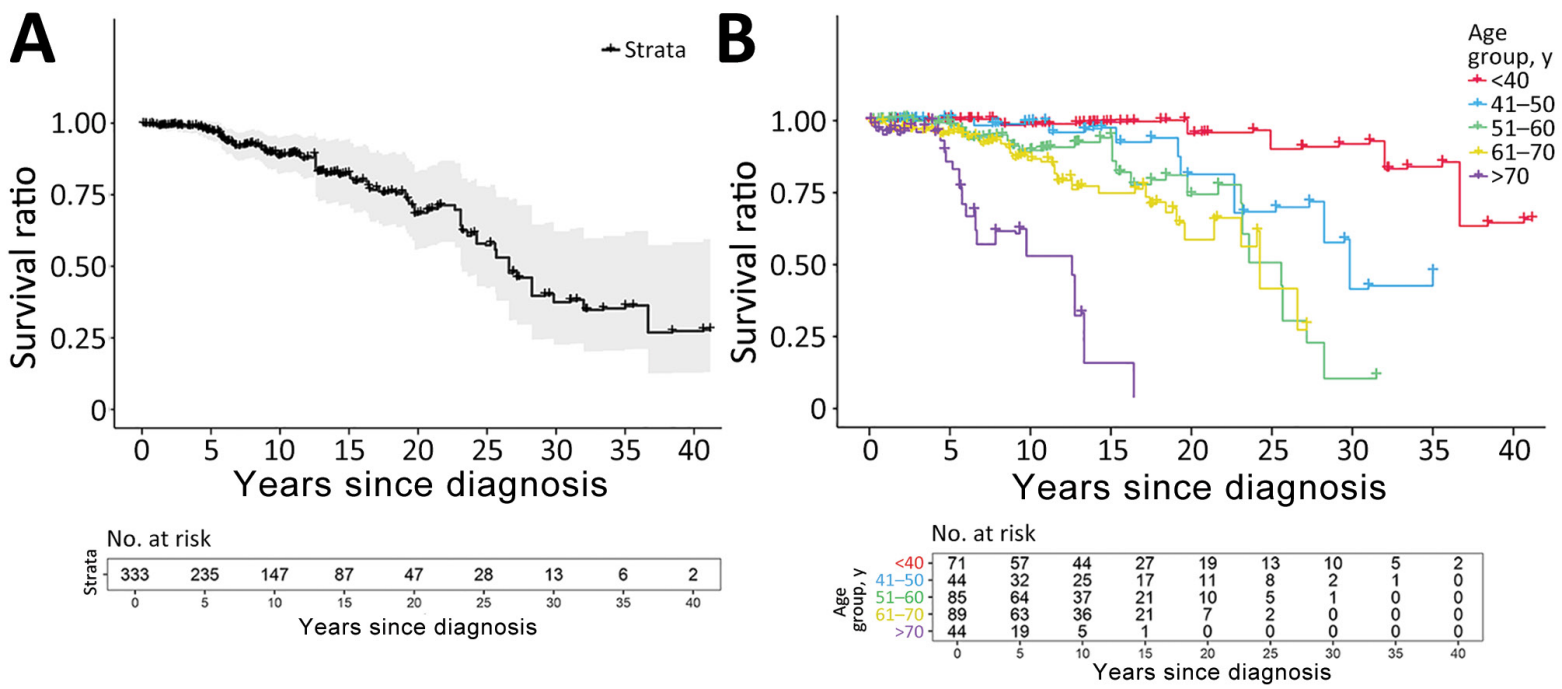
Relative survival analysis of alveolar echinococcosis cases, University Hospital Zurich, Zurich, Switzerland, 1973–2022. A) Relative survival of alveolar echinococcosis patients compared with the population of Switzerland. B) Relative survival grouped by age at alveolar echinococcosis diagnosis. One patient with an alveolar echinococcosis diagnosis at 86 years of age and died at age 99 was excluded for better visualization (Appendix Figure 2).

Relative survival of our cohort of AE patients compared with the population of Switzerland started with a survival ratio around 1.0 but decreased over time (Figure 5). The survival ratio was higher in younger compared with the elderly AE patients; in particular, patients <40 years of age demonstrated a survival ratio of 1.0 over 20 years after diagnosis (Figure 5). Of note, those figures leave out 1 patient who had AE diagnosed in 2008 at 86 years of age (above the average life expectancy for men in Switzerland) and died at 99 years of age, resulting in a bump in the relative survival curves after 13 years (Appendix Figure 2). In the additive relative survival model, only age at diagnosis was significantly associated with overall survival (Table 4). AE-specific variables, in particular stage of the disease but also treatment initiation (surgery or benzimidazole drug treatment) within 1 year, did not show significantly associated coefficient estimates (Table 4).

**Table 4:** Parameters of the relative survival analysis by using an additive model for cases of alveolar echinococcosis, University Hospital Zurich, Zurich, Switzerland, 1973–2022*

Characteristic	Estimate	95% CI	p value
Age at diagnosis	0.078	0.05–0.11	<0.0001
Year of diagnosis	−0.018	−0.04 to 0.00	0.11
Sex, male vs. female	0.033	−0.47 to 0.53	0.90
Stage	−0.036	−0.29 to 0.22	0.78
Surgery within 1 year	−0.411	−0.93 to 0.10	0.12
Benzimidazole within 1 year	−0.514	−1.13 to 0.10	0.10

*The additive survival model provides covariate estimates that reflect the direct effect of each covariate on the excess risk. Compared with a Cox proportional hazard, this effect is absolute and not exponential.

**Figure 6:**
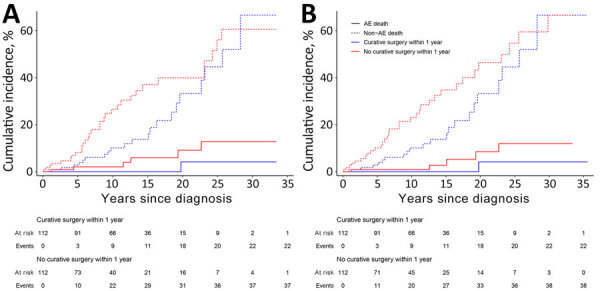
Matched survival analysis of AE cases, University Hospital Zurich, Zurich, Switzerland, 1973–2022. A) Results of nearest neighbor matching. Non-AE death hazard ratio (HR) = 0.48 (95% CI 0.30–0.77), p = 0.002; AE death HR = 0.15 (95% CI 0.02–1.27), p = 0.082. B) Results after genetic matching. Non-AE death HR = 0.49 (95% CI 0.29–0.84), p = 0.009; AE death HR = 0.18 (95% CI 0.02–1.56), p = 0.12. Patients undergoing curatively intended surgery within 1 year of diagnosis showed better overall survival. AE-related death did not differ after matching. AE, alveolar echinococcosis.

Before propensity score matching, baseline characteristics of patients undergoing curatively intended surgery within 1 year were considerably different from the remaining patients (curatively intended surgery after 1 year, palliative surgery, or medical treatment only) (Appendix Table 6). Both 1:1 nearest neighbor and 1:1 genetic matching resulted in 112 matched pairs of patients, including all patients with curative surgery within 1 year (all treated patients), with much better balance of baseline characteristics (Appendix Tables 7, 8). The absolute standardized mean difference was considerably reduced for all characteristics and was <0.2 after matching, except for age at AE diagnosis and overall distance (Appendix Figure 3). In both matched analyses, patients with curative surgery within 1 year showed a better overall survival, with a hazard ratio (HR) of 0.48 (95% CI 0.30–0.77) for 1:1 nearest neighbor matching and 0.49 (95% CI 0.29–0.84) for 1:1 genetic matching (Figure 6, panels A, B). Regarding the occurrence of AE death, matching resulted in an even lower number of events (n = 7 with 1:1 nearest neighbor matching, n = 6 with genetic matching) than observed in the whole cohort (n = 13). Curative surgery within 1 year was not associated with a significant disease-specific survival benefit (HR 0.15 [95% CI 0.02–1.27] after nearest neighbor matching; HR 0.18 [95% CI 0.02–1.56] after genetic matching) (Figure 6, panels A, B).

## Discussion

Our study demonstrates major changes in the clinical manifestations and treatment of AE patients over a 50-year period in Zurich, Switzerland. In addition, survival was mainly limited by non-AE causes, and early curative surgery did not provide a survival benefit in our cohort.

Historically, AE prognosis depended on complete surgical removal of all parasitic tissue. The 2010 World Health Organization guidelines recommend radical resections for all suitable patients (*16*). Despite a shift toward earlier AE stages, our cohort did not show an expected rise in curative surgeries, especially in the last decade of the study period, which might be explained by 2 factors. First, incidentally diagnosed AE cases were more frequently considered inactive, and patients were more likely to refuse surgery. Second, whereas many patients were considered inoperable because of disease extent, the perception of operability or willingness to perform more extensive operations and risk incomplete (R1) resection could have changed over the decades because of the increasingly positive experience with benzimidazole therapy in inoperable patients.

We made several observations about patients who died from AE. Most of those cases were diagnosed before 2000 and in an advanced stage with biliary or vascular complications. For patients who had palliative surgery, it is unclear whether complications leading to death were because of the disease or the surgery itself; only 1 patient in the entire cohort met the criteria for surgical death, defined as any death within 30 days or during the same hospitalization (*27*). After an in-house study, University Hospital Zurich stopped performing palliative resections, and now its care providers would not consider surgery for most patients (*17*). University Hospital Zurich has also become more restrictive concerning biliary tract interventions because of the risk for infectious complication (*20*). Despite receiving mainly palliative or no surgery, the median survival of patients who died of AE was 159 months, highlighting the effectiveness of benzimidazole drugs in slowing disease progression.

Relative survival analysis of AE patients compared with the population of Switzerland revealed several key findings. Unlike a cohort in France that reported excess death in the first 2 years after diagnosis (*23*), our analysis showed a steady decline in relative survival starting 5 years after diagnosis. The decline was age dependent; older patients (>70 years of age at AE diagnosis) showed an earlier decline of relative survival, whereas younger patients (<40 years of age at AE diagnosis) showed similar survival to the general population over a very long time. Disease-specific factors, such as stage and year of diagnosis or treatment within 1 year, were not linked to survival, contrasting with the France study (*23*). That finding might be because most of the patients in this study received timely benzimidazole drug treatment. However, direct comparison is limited because of the absence of baseline treatment data in the France study (*23*). Our findings suggest AE is not the main determinant of life expectancy in infected patients, and the gradual decline of relative survival 5 years after diagnosis could reflect a generally sicker population.

To analyze the effect of early curative surgery on the outcome of our patients, we used propensity-score matching, focusing on AE-specific variables related to treatment and outcomes to avoid overfitting the model. Because perception of operability varies among surgeons, we used the PNM classification as a proxy for operability (*14*,*28*). Palliative surgery patients remained in the control group because of their benefit from benzimidazole drug therapy, despite surgery potentially contributing to death in some cases. Whereas our approach may overestimate operability in the control group, the analysis showed no disease-specific survival benefit for early curative surgery performed within 1 year of diagnosis. The analysis is, however, limited by the small number of AE-related deaths in our cohort and the long survival times of patients who died from AE. The difference in overall survival between groups likely reflects residual confounding, no differences were observed for non-AE causes of death.

Finally, we confirm previous observations of rising AE cases since 2000 and great improvement of patient survival in patients with inoperable disease (*3*,*4*,*19*). In our cohort, the rise of annual AE cases was accompanied by a substantial increase in incidental findings and a shift toward earlier stages, although symptomatic patients with an advanced disease stage remained the majority. This difference might be because of advances in classification of lesions on imaging and histopathology (*29*–*31*). Contrary to expectation, patients with incidental findings were on average not younger but older at diagnosis. This finding has 2 implications. First it eliminates the possibility of a lead-time bias in our survival analysis. Second, the speculation arises if some of those patients would ever have become symptomatic or suffered complications or death because of AE.

In conclusion, our study shows that because of the excellent disease control with benzimidazole drug therapy, curatively intended surgery is only associated with a marginal disease-specific survival benefit. Those findings give reason to change our perception of optimal medical care in AE patients. Today, treatment decisions should be made on the basis of the patient’s expected remaining years with the disease and the potential complications and cost-effectiveness of either a surgical or conservative approach. Although younger patients will most likely benefit from radical resection, older patients may not.

Appendix Additional information about comprehensive survival analysis of alveolar echinococcosis patients, university hospital Zurich, 1973–2022.
