# Supplementary material for: Comprehensive Survival Analysis of Alveolar Echinococcosis Patients, University Hospital Zurich, Zurich, Switzerland, 1973–2022
Source: Emerg Infect Dis. 2025 May;31(5):906–16. doi: 10.3201/eid3105.241608 (PMC12044251; doi:10.3201/eid3105.241608)
Supplement: Appendix — Additional information about comprehensive survival analysis of alveolar echinococcosis patients, university hospital Zurich, 1973–2022. [file 24-1608-Techapp-s1.pdf]

Article DOI: https://doi.org/10.3201/eid3105.241608

EID cannot ensure accessibility for supplementary materials supplied by authors. Readers who have difficulty accessing supplementary content should contact the authors for assistance.

Comprehensive Survival Analysis of Alveolar Echinococcosis Patients, University Hospital Zurich, 1973–2022

Appendix.

Appendix Table 1. PNM Classifications and site of involved organs

Variable	Cohort
n total	334
PNM Classification, n (%)	
P	
X	7 [2.1]
0	3 [0.9]
1	132 [39.5]
2	56 [16.8]
3	75 [22.5]
4	61 [18.3]
N	
X	13 [3.9]
0	225 [67.4]
1	96 [28.7]
M	
X	78 [23.4]
0	215 [64.4]
1	41 [12.3]
Neighboring organ involved, n (%)	
Diaphragm	44 [13.2]
Retroperitoneum/Pancreas/Kidney/Adrenal gland	37 [11.1]
Abdominal/chest wall	32 [9.6]
Pleura/Lung	13 [3.9]
Abdominal cavity	8 [2.4]
Pericard/Heart	7 [2.1]
Site of distant metastasis, n (%)	
Lung	20 [6.0]
Brain	8 [2.4]
Abdominal cavity	8 [2.4]
Spleen	7 [2.1]
Kidney	2 [0.6]
Skeleton	2 [0.6]
Other	2 [0.6]

Other metastasis: Adrenal gland, subcutis

Appendix Table 2. Observed clinical course of AE patients

Clinical course	Cohort
n total	334
Follow-up time in months, median (IQR)	104.5 [51.25, 181]
Cured, n (%)	100 [29.9]
Not cured, n (%)	234 [70.1]
Recurrence after curatively-intended surgery, n (%)	13 [3.9]
Progression events*, n (%)	20 [6.0]
recurrent or new onset cholestasis +/- cholangitis	12 [3.6]
ascites/variceal bleeding	4 [1.2]
cyst rupture / cyst infection / fistula formation	5 [1.5]
Structured BMZ treatment discontinuation, n (%)	28 [8.5]
BMZ treatment duration, median (IQR)	58.5 [42.75, 91.25]
BMZ discontinuation due to other reasons, n (%)	33 [9.9]
TRAE	10 [3.0]
non-structured discontinuation (after R1 or R2 resection)	8 [2.4]
terminal illness other than AE	7 [2.1]
patient choice	4 [1.2]
intended pregnancy	2 [0.6]
unknown/other	2 [0.6]

BMZ: benzimidazole, IQR: inter-quartile range, TRAE: treatment related adverse event.
*in palliative- or non-resected patients

Appendix Table 3. Symptomatic versus incidental diagnosis

Variable	Cohort	Symptomatic	Incidental
N	334	201	127
Age at diagnosis in years, median (IQR)	57.5 [44.0, 65.8]	56.0 [41.0, 64.0]	60.0 [52.0, 67.5]
Sex, n (%)			
male	142 [42.5]	82 [40.8]	56 [44.1]
female	192 [57.5]	119 [59.2]	71 [55.9]
AE Stage, n (%)			
I	93 [27.8]	28 [13.9]	63 [49.6]
II	42 [12.6]	22 [10.9]	19 [15.0]
IIIa	50 [15.0]	34 [16.9]	16 [12.6]
IIIb	74 [22.2]	57 [28.4]	17 [13.4]
IV	68 [20.4]	55 [27.4]	12 [9.4]
Unclassified	7 [2.1]	5 [2.4]	0 [0]
Surgical therapy, n (%)			
Time to surgery in months, median (IQR)	151 [45.2]	101 [50.3]	45 [35.4]
Reason to omit surgery	1 [0, 4]	1 [0, 3]	1 [0, 2]
Inactive disease, n (%)	13 [3.9]	0 [0]	13 [10.2]
Patient refused, n (%)	22 [6.6]	5 [2.5]	17 [13.4]
Comorbidity, n (%)	9 [2.7]	2 [1.0]	7 [5.5]
Disease extent, n (%)	137 [41.0]	91 [45.3]	45 [35.4]
Benzimidazole therapy, n (%)	315 [94.3]	200 [99.5]	109 [85.8]
Time to BMZ in months, median (IQR)	1 [0, 2]	2 [0, 4]	1 [0, 4]
Structured treatment discontinuation, n (%)	24 [7.2]	16 [8.0]	7 [5.5]
Follow-up time in months, median (IQR)	104.5 [51.25, 181]	129 [69, 210]	71 [31,131.5]
All death, n (%)	90 [26.9]	63 [31.3]	26 [20.5]
AE-associated death, n (%)	13 [3.9]	13 [6.5]	0 [0]

Six patients with unknown symptoms at diagnosis are not represented in either the symptomatic or incidental group. BMZ: benzimidazole

Appendix Table 4. Descriptive statistics, overall and stratified by decade of diagnosis

Characteristic	Overall	1973–1982	1983–1992	1993–2002	2003–2012	2013–2022
N	334	29	36	38	88	143
YoB (median [IQR])	1952.0 [1941.0, 1962.8]	1922.0 [1917.0, 1935.0]	1936.5 [1921.0, 1947.0]	1940.0 [1934.2, 1954.5]	1953.0 [1944.0, 1965.2]	1959.0 [1950.0, 1971.0]
Age at Dg (years) (median [IQR])	57.5 [44.0, 65.8]	56.0 [42.0, 61.0]	50.0 [39.5, 66.0]	60.0 [47.2, 65.8]	55.0 [40.8, 63.0]	59.0 [48.0, 67.0]
Height (cm) (median [IQR])	167.5 [162.0, 174.8]	162.0 [154.0, 170.5]	164.0 [158.0, 170.0]	167.0 [163.8, 174.2]	168.0 [163.2, 174.8]	170.0 [163.0, 178.0]
Weight (kg) (median [IQR])	66.5 [58.0, 78.2]	59.0 [55.5, 67.5]	61.5 [56.0, 73.5]	65.0 [59.0, 74.0]	71.0 [59.0, 82.0]	67.0 [60.0, 78.0]
BMI (median [IQR])	23.4 [21.2, 26.4]	23.2 [21.8, 24.9]	23.0 [20.7, 26.5]	23.4 [21.1, 26.2]	23.8 [21.5, 26.4]	23.4 [21.3, 26.7]
Sex = male (%)	142 [42.5]	11 [37.9]	13 [36.1]	14 [36.8]	47 [53.4]	57 [39.9]
WHO (%)						
Possible	4 [1.2]	1 [3.4]	0 [0.0]	1 [2.6]	0 [0.0]	2 [1.4]
Probable	144 [43.1]	8 [27.6]	9 [25.0]	14 [36.8]	33 [37.5]	80 [55.9]
Definitive	186 [55.7]	20 [69.0]	27 [75.0]	23 [60.5]	55 [62.5]	61 [42.7]
Discovery of disease (%)						
Symptoms	201 [60.2]	26 [89.7]	30 [83.3]	25 [65.8]	55 [62.5]	65 [45.5]
Abnormal liver values	23 [6.9]	2 [6.9]	2 [5.6]	2 [5.3]	6 [6.8]	11 [7.7]
Incidental finding on imaging	100 [29.9]	1 [3.4]	2 [5.6]	9 [23.7]	26 [29.5]	62 [43.4]
Other	4 [1.2]	0 [0.0]	0 [0.0]	2 [5.3]	0 [0.0]	2 [1.4]
Unknown	6 [1.8]	0 [0.0]	2 [5.6]	0 [0.0]	1 [1.1]	3 [2.1]
Stage (%)						
I	93 [28.4]	2 [7.1]	5 [15.6]	10 [27.0]	21 [24.1]	55 [38.5]
II	42 [12.8]	5 [17.9]	3 [9.4]	3 [8.1]	14 [16.1]	17 [11.9]
IIIa	50 [15.3]	1 [3.6]	6 [18.8]	4 [10.8]	19 [21.8]	20 [14.0]
IIIb	74 [22.6]	8 [28.6]	12 [37.5]	12 [32.4]	20 [23.0]	22 [15.4]
IV	68 [20.8]	12 [42.9]	6 [18.8]	8 [21.6]	13 [14.9]	29 [20.3]
Surgery aim (%)						
Curative	126 [37.7]	9 [31.0]	17 [47.2]	18 [47.4]	49 [55.7]	33 [23.1]
Palliative	25 [7.5]	10 [34.5]	10 [27.8]	4 [10.5]	1 [1.1]	0 [0.0]
no surgery	183 [54.8]	10 [34.5]	9 [25.0]	16 [42.1]	38 [43.2]	110 [76.9]
Surgery timing (%)						
within 1 year	140 [41.9]	16 [55.2]	26 [72.2]	18 [47.4]	49 [55.7]	31 [21.7]
between 1 and 5 years	8 [2.4]	3 [10.3]	0 [0.0]	3 [7.9]	0 [0.0]	2 [1.4]
later than 5 years	3 [0.9]	0 [0.0]	1 [2.8]	1 [2.6]	1 [1.1]	0 [0.0]
Benzimidazole timing (%)						
within 1 year	298 [89.2]	21 [72.4]	34 [94.4]	34 [89.5]	83 [94.3]	126 [88.1]
between 1 and 5 years	9 [2.7]	2 [6.9]	2 [5.6]	2 [5.3]	0 [0.0]	3 [2.1]
later than 5 years	8 [2.4]	6 [20.7]	0 [0.0]	0 [0.0]	1 [1.1]	1 [0.7]
no therapy	19 [5.7]	0 [0.0]	0 [0.0]	2 [5.3]	4 [4.5]	13 [9.1]

YoB = Year of birth, Age at Dg = Age at diagnosis, WHO = WHO diagnosis criteria

Appendix Table 5. Characteristics of patients with unknown cause of death

ID	YoD	Age	Sex	Stage	Sympt.	Compl.	Surgery	Aim	BMZ	Follow-up time	Age at death	Last disease status
14	1975	34	m	IV	Yes	biliary	yes	palliative	yes	440	79	SD 2.5 years prior to death; in nursing home due to dementia
15	1977	51	m	IV	Yes	none	yes	curative	yes	339	80	SD 1 year prior to death
16	1977	42	m	IV	Yes	none	yes	palliative	yes	339	71	SD 1 year prior to death
17	1979	53	f	I	No	none	yes	curative	yes	184	93	recurrence, SD 15 years prior to death
18	1981	64	f	IV	Yes	none			yes	291	89	SD 1 year prior to death
19	1982	70	f	IIIb	Yes	none	yes	palliative	yes	107	93	SD 14 years prior to death
20	1987	66	f	IV	Yes	none	yes	palliative	yes	319	93	SD 5 years prior to death
21	1988	71	f	IIIa	No	none			yes	197	91	SD 4 years prior to death
22	1989	44	f	IIIb	Yes	none	yes	curative	yes	78	70	no recurrence 20 years prior to death
23	1996	56	m	IV	Yes	unknown	yes	curative	yes	283	81	no recurrence 1.5 years prior to death
24	2002	64	m	IIIb	Yes	none			yes	206	82	SD 5 months prior to death
25	2004	78	m	IIIa	Yes	none	yes	curative	yes	117	96	recurrence, SD 8 years prior to death
26	2008	86	f	IIIb	Yes	none			yes	49	99	SD 9 years prior to death

Yod = Year of diagnosis, Age = Age at diagnosis (in years), m = male, f = female, Sympt. = Symptoms at Diagnosis, Compl. = Complications present at diagnosis, BMZ: benzimidazole therapy, Follow-up time (months), SD = stabile disease

Appendix Table 6. Explanatory variables for propensity score matching

Variable	Overall	Early curative surgery	No early curative surgery
N	331	118	213
Year of diagnosis, mean (SD)	2006.37 (13.47)	2004.39 (12.25)	2007.47 (14.00)
Age at diagnosis, mean (SD)	54.54 (15.03)	50.90 (14.87)	56.56 (14.78)
P, n (%)			
1	132 (40.7)	64 (57.1)	68 (32.1)
2	56 (17.3)	25 (22.3)	31 (14.6)
3	75 (23.1)	16 (14.3)	59 (27.8)
4	61 (18.8)	7 (6.2)	54 (25.5)
X or missing	7 (2.1)	6 (5.1)	1 (0.3)
N, n (%)			
0	225 (69.4)	86 (76.8)	139 (65.6)
1	96 (29.6)	25 (22.3)	71 (33.5)
X or missing	10 (3.0)	7 (5.9)	3 (1.4)
M, n (%)			
0	286 (86.4)	102 (86.4)	184 (86.5)
1	38 (11.7)	10 (8.9)	28 (13.2)
Missing	7 (2.1)	6 (5.1)	1 (0.3)
Incidental finding, n (%)	126 (38.1)	39 (33.1)	87 (40.8)
Complication at diagnosis, n (%)	60 (18.1)	17 (14.4)	43 (20.2)
BMZ start ≤ 12 months, n (%)	296 (89.4)	109 (92.4)	187 (87.8)

Three patients with non-hepatic disease omitted.

Appendix Table 7. Covariate balance after nearest neighbor matching

Variable	Overall	Early curative surgery	No early curative surgery	Missing
N	224	112	112	
Year of diagnosis, mean (SD)	2005.71 (13.47)	2005.04 (12.10)	2006.38 (14.74)	0.0
Age at diagnosis, mean (SD)	53.69 (14.49)	51.71 (14.59)	55.67 (14.18)	0.0
P, n (%)				0.0
1	119 (53.1)	64 (57.1)	55 (49.1)	
2	50 (22.3)	25 (22.3)	25 (22.3)	
3	40 (17.9)	16 (14.3)	24 (14.3)	
4	15 (6.7)	7 (6.2)	8 (7.1)	
N, n (%)				0.0
0	170 (75.9)	86 (76.8)	84 (75.0)	
1	52 (23.2)	25 (22.3)	27 (24.1)	
X	2 (0.9)	1 (0.9)	1 (0.9)	
M, n (%)				0.0
1	23 (10.3)	10 (8.9)	13 (11.6)	
Incidental finding, n (%)	83 (37.1)	39 (34.8)	44 (39.3)	0.0
Complication at diagnosis, n (%)	36 (16.1)	16 (14.3)	20 (17.9)	0.0
BMZ start ≤ 12 months, n (%)	208 (92.9)	104 (92.9)	104 (92.9)	0.0

Appendix Table 8. Covariate balance after genetic matching

Variable	Overall	Early curative surgery	No early curative surgery	Missing
N	224	112	112	
Year of diagnosis, mean (SD)	2005.81 (13.10)	2005.04 (12.10)	2006.57 (14.03)	0.0
Age at diagnosis, mean (SD)	53.56 (13.98)	51.71 (14.59)	55.41 (13.16)	0.0
P, n (%)				0.0
1	123 (54.9)	64 (57.1)	59 (52.7)	
2	48 (21.4)	25 (22.3)	23 (20.5)	
3	35 (15.6)	16 (14.3)	19 (17.0)	
4	18 (8.0)	7 (6.2)	11 (9.8)	
N, n (%)				0.0
0	167 (74.6)	86 (76.8)	81 (72.3)	
1	55 (24.6)	25 (22.3)	30 (26.8)	
X	2 (0.9)	1 (0.9)	1 (0.9)	
M, n (%)				0.0
1	23 (10.3)	10 (8.9)	13 (11.6)	
Incidental finding, n (%)	85 (37.9)	39 (34.8)	46 (41.1)	0.0
Complication at diagnosis, n (%)	36 (16.1)	16 (14.3)	20 (17.9)	0.0
BMZ start ≤ 12 months, n (%)	203 (90.6)	104 (92.9)	99 (88.4)	0.0

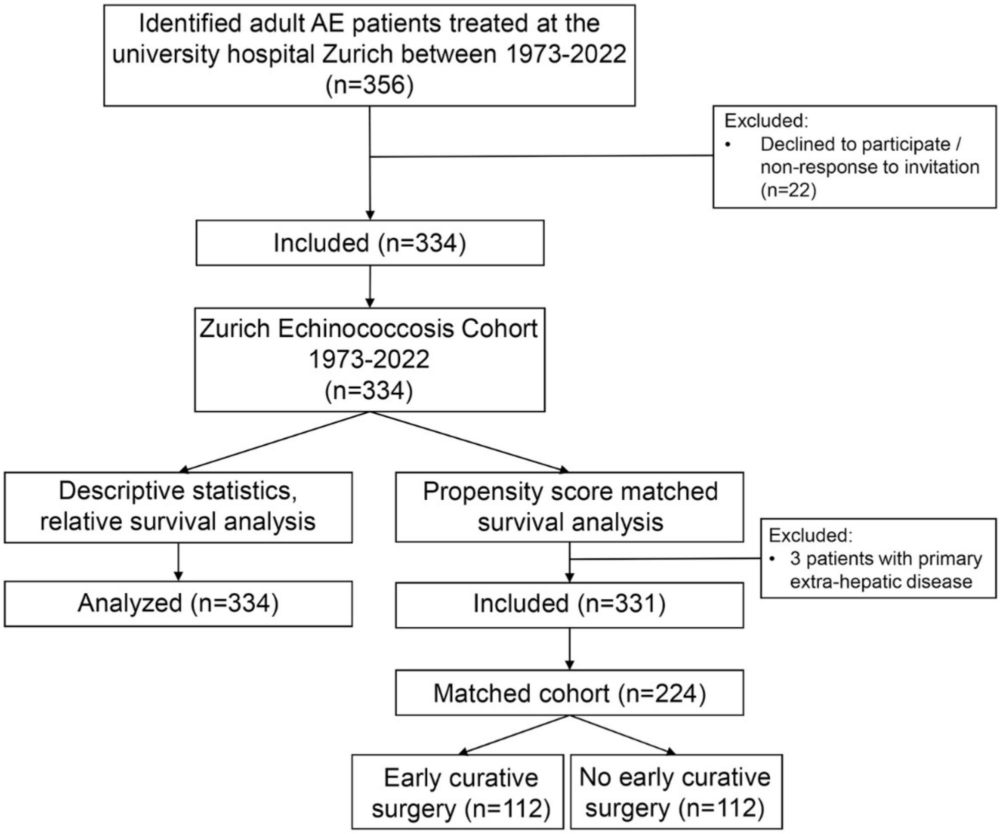

Appendix Figure 1. Study Flow Diagram

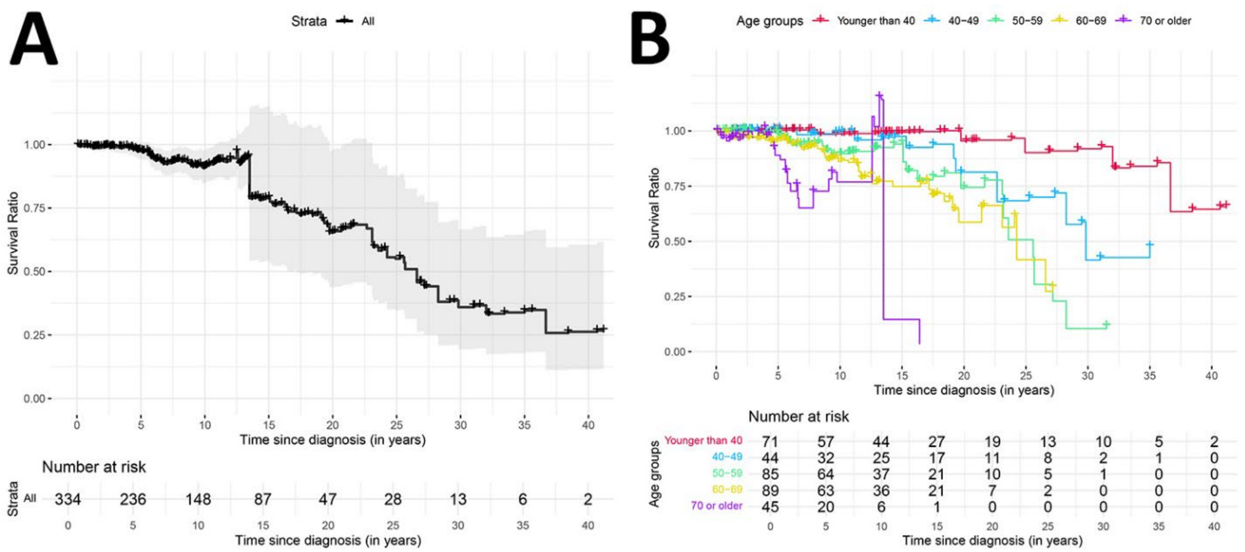

Appendix Figure 2. Relative survival including the patient, who was diagnosed at age 86 and died at age 99.

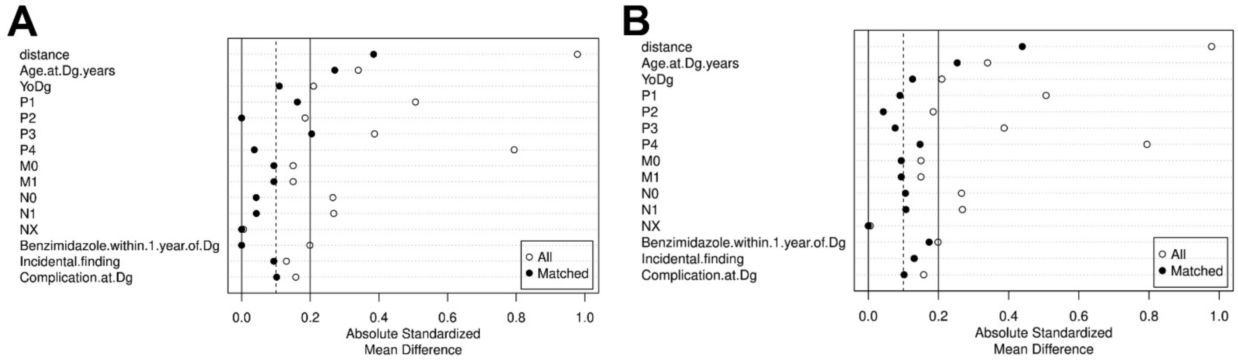

Appendix Figure 3. Covariate balance before and after propensity score matching. A) Covariate balance of nearest neighbor matching. B) Covariate balance of genetic matching.
